# The effect of mesenchymal stromal cell sheets on the inflammatory stage of flexor tendon healing

**DOI:** 10.1186/s13287-016-0406-0

**Published:** 2016-09-27

**Authors:** Hua Shen, Ioannis Kormpakis, Necat Havlioglu, Stephen W. Linderman, Shelly E. Sakiyama-Elbert, Isaac E. Erickson, Thomas Zarembinski, Matthew J. Silva, Richard H. Gelberman, Stavros Thomopoulos

**Affiliations:** 1 Department of Orthopaedic Surgery, Washington University, 660 South Euclid, Campus, Box 8233, St. Louis, MO 63110 USA; 2 Department of Biomedical Engineering, Washington University in St. Louis, St. Louis, MO USA; 3 Department of Pathology, John Cochran VA Medical Center, St. Louis, MO USA; 4 Biotime, Inc., Alameda, CA USA; 5 Department of Orthopedic Surgery, Columbia University, Black Building 1408, 650 W 168 ST, New York, NY 10032 USA; 6 Department of Biomedical Engineering, Columbia University, New York, NY USA

**Keywords:** Adipose-derived mesenchymal stromal cells, Cell sheet, Tendon, Tissue engineering, Intrasynovial tendon, Flexor tendon, Inflammation, Macrophage

## Abstract

**Background:**

The clinical outcomes following intrasynovial flexor tendon repair are highly variable. Excessive inflammation is a principal factor underlying the formation of adhesions at the repair surface and affecting matrix regeneration at the repair center that limit tendon excursion and impair tendon healing. A previous in-vitro study revealed that adipose-derived mesenchymal stromal cells (ASCs) modulate tendon fibroblast response to macrophage-induced inflammation. The goal of the current study was therefore to explore the effectiveness of autologous ASCs on the inflammatory stage of intrasynovial tendon healing in vivo using a clinically relevant animal model.

**Methods:**

Zone II flexor tendon transections and suture repairs were performed in a canine model. Autologous ASC sheets were delivered to the surface of repaired tendons. Seven days after repair, the effects of ASCs on tendon healing, with a focus on the inflammatory response, were evaluated using gene expression assays, immunostaining, and histological assessments.

**Results:**

ASCs delivered via the cell sheet infiltrated the host tendon, including the repair surface and the space between the tendon ends, as viewed histologically by tracking GFP-expressing ASCs. Gene expression results demonstrated that ASCs promoted a regenerative/anti-inflammatory M2 macrophage phenotype and regulated tendon matrix remodeling. Specifically, there were significant increases in M2-stimulator (*IL-4*)*,* marker (*CD163* and *MRC1*), and effector (*VEGF*) gene expression in ASC-sheet treated tendons compared with nontreated tendons. When examining changes in extracellular matrix expression, tendon injury caused a significant increase in scar-associated *COL3A1* expression and reductions in *COL2A1* and *ACAN* expression. The ASC treatment effectively counteracted these changes, returning the expression levels of these genes closer to normal. Immunostaining further confirmed that ASC treatment increased CD163^+^ M2 cells in the repaired tendons and suppressed cell apoptosis at the repair site.

**Conclusions:**

This study provides a novel approach for delivering ASCs with outcomes indicating potential for substantial modulation of the inflammatory environment and enhancement of tendon healing after flexor tendon repair.

## Background

Laboratory and clinical research has led to improvements in the outcomes of flexor tendon repair through enhanced suture methods and refined rehabilitation protocols [1, 2]. However, poor healing at the repair site can lead to gapping, adhesion formation, and rupture, severely impeding return of digital function in a significant number of patients [3, 4]. Tendon healing is a complex process consisting of three sequential overlapping stages: inflammation, proliferation, and remodeling [5–7]. Although most reported complications have occurred within the first 3 postoperative weeks [2, 3], recent biologic approaches have focused on the later proliferation and remodeling stages and have shown limited positive effects [8, 9]. Modulation of the early inflammatory response that initiates the repair process may therefore be necessary for a satisfactory recovery of tendon function.

The inflammatory cascade following tendon injury and repair is dynamic and complex. Within the first 2–3 days, gene expression levels of proinflammatory cytokines increase by many thousand-fold at the wound site [6]. Such increases are accompanied by the infiltration of large numbers of inflammatory cells, including monocytes and neutrophils [6]. The initial postrepair cellular response, which often leads to adhesion formation, matrix degradation, and cell death [6, 7, 10], is largely detrimental to tendon healing. Broad attempts to suppress inflammation after tendon repair, however, may prevent the recruitment of regenerative fibroblasts (FBs) and impair the tendon healing process. Careful modulation of the inflammatory cascade is therefore necessary to improve tendon healing, specifically by suppressing negative features such as tendon cell death and matrix degradation and by enhancing positive features associated with tendon cell proliferation and matrix synthesis.

The primary inflammatory cells that infiltrate the repair site during flexor tendon healing are macrophages, which are derived from monocytes [6]. Depending on their local environment, macrophages acquire distinct phenotypes, which may be classified into two major categories: a proinflammatory type 1 (M1) and an anti-inflammatory type 2 (M2) phenotype [11]. Although M1 macrophages are necessary for the clearance of damaged tissue and the initiation of healing response, they also produce the proinflammatory cytokines IL-1, TNF-α, and IL-6 as well as cytotoxic reactive oxygen and nitrogen intermediates. Collectively, these factors induce inflammation, scar formation, cell death, and matrix degradation, thus impeding tendon healing. In contrast, M2 macrophages produce anti-inflammatory IL-1 receptor antagonist (IL-1RA) and IL-10, growth factors, and extracellular matrix, thus mitigating inflammation and scarring and facilitating matrix remodeling and wound healing [11]. The M1 macrophages are reported to dominate the early inflammatory stage of tendon healing, while the M2 macrophages are mainly detected in the latter remodeling stage [12, 13]. Promotion of the M2 phenotype during the earliest stages of tendon healing may therefore limit harmful inflammation, protecting both tendon cells and matrix, and facilitating tissue regeneration.

The M2 phenotype may be induced through the immunoregulatory function of adipose-derived mesenchymal stromal cells (ASCs). In addition to their easy accessibility and tenogenic potential [14], we recently reported that ASCs protect tendon fibroblasts (TFs) from the negative effects of M1 macrophages by inducing an M1 to M2 phenotypic switch in vitro [15]. Others have shown, in a cutaneous wound healing model, that mesenchymal stromal cells (MSCs) derived from gingiva consistently induce the M2 phenotype and improve healing [16]. Nevertheless, whether undifferentiated ASCs can regulate the macrophage phenotype and ultimately improve flexor tendon healing in vivo remains unclear. The goal of this study was therefore to evaluate the impact of autologous ASCs on the early inflammatory stage of flexor tendon healing. The hypothesis of this study was that autologous ASCs, delivered to the repair surface, would infiltrate the repair site, improve matrix remodeling, and facilitate tendon healing via promoting macrophages toward the M2 phenotype and therefore away from the M1 phenotype.

## Methods

### Autologous ASC sheet preparation and in-vitro characterization

**Fig. 1:**
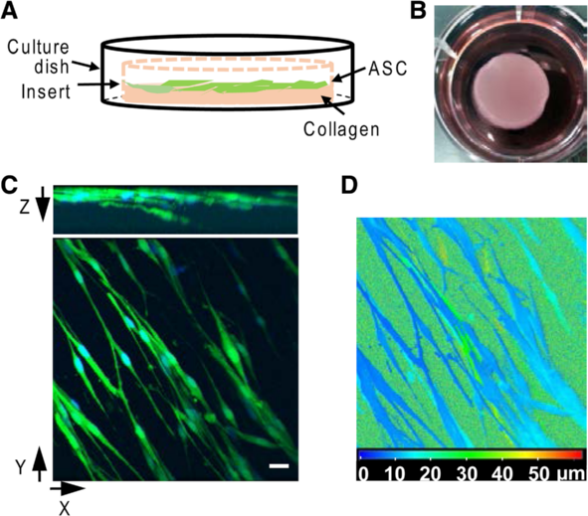
Generation of ASC sheet. **a** Schematic illustration of ASC sheet preparation. **b** Representative image of an ASC sheet in a well of a six-well plate. **c** 3-D projection of an ASC sheet, revealing the GFP-expressing ASCs (*green*) in the collagen matrix. Cell nuclei were counterstained with bisBenzimide H 33258 (*blue*). *Bar* = 20 μm. **d** Depth-coded image of (**c**). *Blue* and *red* color scale indicates the closest and furthest, respectively, from the surface of the ASC sheet. *ASC* adipose-derived mesenchymal stromal cell

Subcutaneous fat was obtained 2 weeks prior to tendon repair for ASC isolation and subsequent cell sheet preparation. ASC isolation and culture was performed as described previously [14]. To prepare ASC sheets, 1 ml of collagen solution (pH 7.2), containing 2 mg/ml type I collagen from rat tail tendon (Corning Life Sciences, Bedford, MA, USA), was added to a ring-shaped silicone insert (Ø 19 mm) placed in a well of a six-well plate and incubated at 37 °C for 1 h to gel. The gelled collagen was equilibrated overnight in α -MEM (Mediatech, Inc., Manassas, VA, USA) containing 10 % fetal bovine serum (Hyclone Laboratories, Logan, UT, USA) and 100 U/ml penicillin–steptomycin (Life Technologies, Gaithersburg, MD, USA). Passage 3 ASCs were then plated on top of the collagen layer at a density of 10,000 cells/cm^2^. The resulting cell sheet was cultured for 3–4 days in vitro with medium change every other day (Fig. 1a, b). To track ASCs in vitro and in vivo, some cell sheets were prepared with ASCs pretransduced with green fluorescent protein (GFP)-expressing lentivirus.

The viability of ASCs in the cell sheet was examined after 5 days in culture by staining live cell sheets with propidium iodide (PI; Life Technologies) according to the manufacturer’s instructions. Phase-contrast and fluorescent images were taken at four different areas of each stained cell sheet (*N* = 3 cell isolations). The numbers of total and PI-positive cells at each of the four areas were quantified and averaged. Cell viability for each sample was calculated as the ratio of total cell count minus PI-positive cell count, divided by total cell count, and presented as a percentage. To estimate the population doubling time of the ASCs, cell sheets cultured for 7 days were fixed with 4 % paraformaldehyde (PFA) in phosphate-buffered saline (PBS) for 15 min and then stained with bisBenzimide H 33258 (Sigma-Aldrich, St. Louis, MO, USA). The total number of cells within the cell sheets was determined by counting the stained nuclei. The population doubling time of each sample (*N* = 3 cell isolations) was subsequently calculated according to initial cell counts and culture duration.

To determine the cell distribution within ASC sheets, GFP-expressing ASC sheets were fixed in 4 % PFA for 15 min. After three washes with PBS, the ASC sheets were mounted on a coverslip. Z-stack images were obtained under a confocal microscope (Zeiss LSM 5 PASCAL) equipped with a 40×/1.4 oil immersion lens. 3-D images were generated with Zeiss LSM Image Browser 4.2.0.121.

### Development of surgical methods for ASC sheet implantation

A cell delivery system was developed to overcome a number of challenges associated with application of ASCs to repaired flexor tendons. Intrasynovial tendons have: small cross-sectional areas (limiting the volume of material that can be delivered); a high density of extracellular matrix (limiting the diffusion of factors and/or infiltration of cells); and a special need for local delivery (promoting healing at the repair site without concurrent promotion of adhesions between the tendon surface and its sheath). Because prior efforts to apply ASCs to flexor tendon using polymer-based scaffolds inserted into slits within the tendon stumps had negative effects on tendon healing [8]*,* a new cell sheet delivery system was developed.

The ASC sheet did not readily adhere to the flexor tendon surface, so a series of cadaver studies were performed to develop methods for implantation of the ASC sheet during surgical repair. Hyaluronan-based materials can suppress adhesions at the flexor tendon surface and improve tendon gliding [17, 18]. Therefore, a hydrogel consisting of thiol-modified hyaluronan (HA; Biotime, Inc, Alemeda, CA, USA [19]) was used to immobilize the ASC sheet on the tendon surface. The hydrogel was crosslinked in situ with polyethylene glycol diacrylate. PBS was chosen over Ringer’s solution as the gelling buffer to obtain consistent gelation time. With the buffer, approximately 5 min of gelation time resulted in an appropriate viscosity for application of the hydrogel to the tendon surface, and an additional 5 min was necessary to ensure complete gelation and proper adhesion of the hydrogel to the tendon. Three cadaver flexor tendon repairs were performed with application of ASC sheets onto the repair site followed by a layer of HA. After gelation, both synovial sheath and skin were closed and passive motion was applied to the paw to mimic the rehabilitation protocol performed in live animals. After 24 hours, the repaired tendons were exposed, visually examined, and dissected for histological assessment. In all cases, ASC sheets remained over the repair site covered in the hydrogel. Moreover, the implanted cells migrated from the cell sheet wrapping around the tendon surface to the gap between the ends of the severed tendon stumps and within the tendon substance.

### In-vivo animal model

**Fig. 2:**
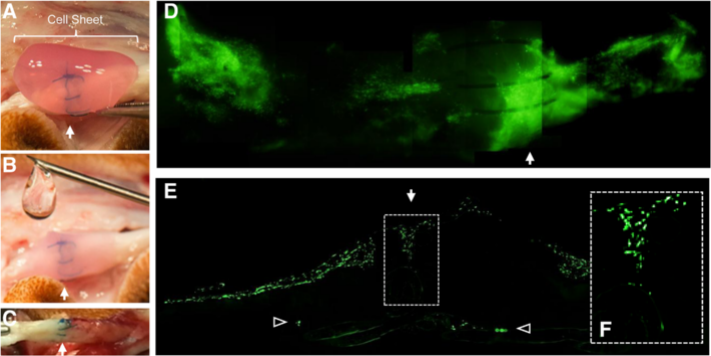
Application of an ASC sheet in tendon repair. **a**, **b** Representative photographs showing the process of applying **a** an ASC sheet and **b** HA to a sutured FDP tendon. **c** Representative photograph showing a FDP tendon 7 days after ASC sheet repair. **d** Representative fluorescent image of a whole-mount FDP tendon repaired with a GFP-expressing ASC sheet. **e**, **f** Representative fluorescent image of a longitudinal section of a FDP tendon repaired with a GFP-expressing ASC sheet. **f** Enlarged image of the boxed region in **e**. *Arrows*, site of tendon repair; *open arrowheads*, GFP-expressing cells in the tendon substance

A clinically relevant canine animal model of intrasynovial flexor tendon injury and repair was used in 12 female adult mongrel dogs (20–30 kg) [20]. Mature animals were chosen to obtain adult ASCs. Tendon transection and repair were performed as described previously in Zone II of the second and the fifth flexor digitorum profundus (FDP) tendons of the right front paw of each animal [6, 9]. After two washes with sterile saline, an autologous ASC sheet was wrapped around a repaired tendon with the cell side facing the tendon (Fig. 2a) and secured in place using HA (Fig. 2b). Five animals received ASC sheets in both repaired digits (+ ASC sheet group). One digit was used for histological study and the other digit was used for gene expression analysis. The remaining seven animals received HA alone in one of the repaired digits (+ HA group) and no treatment in the other repaired digits to serve as HA and repair only control groups, respectively. Among the seven animals, two were used for histology, two were used for gene expression analysis, and three were used for both histology and the gene expression analysis. The latter was achieved by dividing each repaired tendon longitudinally into two parts along the sagittal plane. The corresponding uninjured contralateral digits of all animals served as normal healthy controls. All repaired digits received controlled passive mobilization starting 24 hours after surgery as described previously [6, 9]. Animals were euthanized 7 days after repair.

### RNA isolation and real-time quantitative RT-PCR

Tendon fragments flanking the repair site (~1 cm) were dissected, diced into small pieces, and then pulverized with a Mikro-Dismembrator (Sartorius). Total RNA was subsequently isolated with Trizol Reagent (Life Technologies, Carlsbad, CA, USA) and RNeasy Mini Spin Columns (Qiagen Sciences, Germantown, MD, USA), as described previously [14]. The resulting total RNA (500 ng) was reverse-transcribed into first-strand cDNA using a SuperScript VILO cDNA Synthesis Kit (Life Technologies), according to the manufacturer’s instructions. An initial survey for changes in gene expression was performed with TaqMan real-time PCR by Washington University Genome Technology Access Center using the Fluidigm Biomark™ HD system. All TaqMan primers and probes used in this study were purchased from Applied Biosystems (Table 1). The relative abundances of target genes in repaired digits were analyzed with the comparative Ct (2^–ΔΔCt^) method using *GAPDH* and *PPIB* as endogenous reference genes and compared with those of corresponding normal digits. A follow-up gene expression study was performed to examine macrophage phenotype using SYBR green-based real-time PCR. The relative abundance of target genes in repaired digits was analyzed with the 2^–ΔΔCt^ method using *GAPDH* as the endogenous reference gene and compared with those of corresponding normal digits. A maximum Ct value of 40 was assigned to nondetectable samples. The SYBR green PCR primers of all target genes were purchased from Qiagen (Table 2), except for *SCX* whose primers have been described previously [14].

**Table 1:** TaqMan primer and probes used in the study

Gene symbol	Assay number	RefSeq accession number	Amplicon length (bp)
*ACAN*	Cf02674826_m1	NM_001113455.1	125
*CD163*	Cf02627309_m1	NM_001048020.1	78
*COL1A1*	Cf02623126_m1	NM_001003090.1	87
*COL2A1*	Cf02622836_m1	NM_001006951.1	72
*COL3A1*	Cf02631370_m1	XM_535997.2	78
*COL5A1*	Cf02645008_m1	XM_537804.2	77
*DCN*	Cf02628561_m1	NM_001003228.1	78
*GAPDH*	Cf04419463_gH	NM_001003142.1	54
*IL1B*	Cf02671953_g1	NM_001037971.1	78
*IL6*	Cf02624152_g1	NM_001003301.1	80
*MMP1*	Cf02651000_g1	XM_546546.2	63
*MMP3*	Cf02625960_m1	NM_001002967.1	79
*MMP13*	Cf02623587_m1	XM_536598.2	78
*PPIB*	Cf02629556_m1	M_535510.2	78
*PTGS1*	Cf02624717_m1	NM_001003023.1	75
*PTGS2*	Cf02625600_g1	NM_001003354.1	90
*SOX9*	Cf02625134_g1	NM_001002978.1	103
*TGFB1*	Cf02623324_m1	NM_001003309.1	83
*TGFB3*	Cf02625275_m1	XM_849026.1	118
*TNF*	Cf02628236_m1	NM_001003244.4	75
*TNMD*	Cf02665570_m1	XM_538101.2	106
*VEGFA*	Cf02623448_m1	NM_001003175.2	69

**Table 2:** SYBR green primers used in this study

Gene symbol	Catalogue number	RefSeq accession number	Amplicon length (bp)
*GAPDH*	QT00896756	NM_001003142	83
*IFN-G*	QT00897036	NM_001003174	136
*IL10*	QT01119804	NM_001003077	119
*IL13*	QT00898821	NM_001003384	137
*IL1RN*	QT00896189	NM_001003096	89
*IL4*	QT00896931	NM_001003159	93
*MRC1*	QT01361703	XM_544242	90
*NOS2*	QT01118831	NM_001003186	75

### Histology

FDP tendons were pinned with slight tension on corks and fixed in 4 % PFA in PBS overnight. After three washes with PBS, the fixed tissues were examined and imaged under a fluorescence microscope to determine the location of ASC sheets relative to the repair site. Tissues were then either equilibrated with 30 % sucrose in PBS at 4 °C overnight and embedded in Tissue-Tek O.C.T. compound or dehydrated and embedded in paraffin. Serial coronal sections were then prepared. To evaluate the distribution of ASCs delivered via cell sheet in the repaired tendons, sections (8 μm thick) were counterstained with bisBenzimide H 33258 every 100 μm in depth. Additional sections were stained with hematoxylin and eosin (H&E). Histological assessment was performed blindly on H&E-stained sections (one middle section per tissue) by an independent, board-certified pathologist (NH) using a light microscope equipped with a 20× objective. Each section was divided into three regions relative to the repair site: proximal, center, and distal. Each region was graded for overall cellularity, FBs, polymorphonuclear cells (PMNs), mononuclear cells (MNs), and vascularity, as detailed in Table 3.

**Table 3:** Scoring system for histological assessment of FDP tendons

Score	Cellularity/PMN/MN	FB	Vascularity
1	<50 cells per HPF	<5 %	<5 vessels per HPF
2	51–100 cells per HPF	6–50 %	6–10 vessels per HPF
3	101–150 cells per HPF	>50 %	>10 per HPF
4	>150 cells per HPF		

*FDP* flexor digitorum profundus, *PMN* polymorphonuclear cell, *MN* mononuclear cell and macrophage, *FB* fibroblast, *HPF* high-power field under a 20 × objective

### Immunofluorescence staining

Immunofluorescence staining was performed on frozen sections of tendon tissues. After a brief wash with PBS, the sections were permeabilized in 0.5 % Triton X-100 (Sigma-Aldrich) in PBS for 15 min and blocked with 5 % normal donkey serum in PBS containing 0.1 % Triton X-100 for 30 min. The sections were then incubated with either goat anti-CD163 (sc-1879, 1:50 dilution; Santa Cruz Biotechnology, Santa Cruz, CA, USA) or rabbit anti-cleaved caspase-3 (#9661, 1:400 dilution; Cell Signaling Technology, Beverly, MA, USA) antibodies at 4 °C overnight, followed by Dylight 549-conjugated donkey anti-goat or Cy3-conjugated donkey anti-rabbit (1:400 dilution; Jackson ImmunoResearch, West Grove, PA, USA) antibodies for 1 h. After thorough washes, the stained sections were mounted in a mounting medium containing DAPI nuclear staining reagent (Vector Laboratories, Burlingame, CA, USA). Immunostainings without primary antibodies were included in parallel with each experiment to exclude nonspecific signals. The assessment of immunostainings was performed blindly on two sections per sample.

### Statistics

All data are shown as mean ± standard deviation. A one-way analysis of variance (ANOVA) followed by Student–Newman–Keuls’s post-hoc testing (when appropriate) was performed to compare gene expression among the three repair groups. Two-tailed Student’s *t* tests and Mann–Whitney rank sum tests were used to compare the repair and normal groups for normally and nonnormally distributed data, respectively. All statistic analyses were performed using SigmaStat 3.5 (Systat Software, Inc., Chicago, IL, USA). The significance level was set at *P* < 0.05.

## Results

### In-vitro characterization of the ASC sheet

The ASC sheet, generated by culturing ASCs on the top of a layer of type I collagen, was approximately 500 μm thick (Fig. 1a, b). Within the sheet, ASCs formed three to five cell layers within 100 μm from the surface (Fig. 1c, d). The survival rate of ASCs in the sheet was 95.6 ± 0.3 % (*N* = 3) after 5 days in culture. The calculated population doubling time of ASCs in the sheet after 7 days in culture was 1.9 ± 0.4 days (*N* = 3), comparable with that of ASCs in conventional culture [14]. Taken together, the results demonstrated the successful generation of a viable ASC sheet.

### Implantation of ASC sheet in vivo

ASC sheets were wrapped around sutured FDP tendons at the repair site with the cell side facing the tendons (Fig. 2a). Gelling HA was subsequently applied to secure ASC sheets in place (Fig. 2b). After the HA gelled, the synovial sheath and skin were closed with sutures. All ASC sheet-treated tendons were evaluated 7 days after repair. There were no gaps greater than 3 mm and there were no apparent adhesions in any of the repairs at dissection (Fig. 2c).

ASCs were pretransduced with lentivirus to express GFP. Based on the pattern of GFP expression in whole-mount tendons, the implanted ASC sheets were clearly retained at the repair site and remained in contact with the host tendons (Fig. 2d; *N* = 5). Images from longitudinal sections further revealed that the GFP-expressing cells filled the gap spaces between the two ends of transected and repaired tendon stumps (Fig. 2e, f; *N* = 5), with some cells migrating to the tendon substance (open arrows in Fig. 2e; *N* = 5). These results demonstrated that viable cells were successfully delivered to the tendon repair site using ASC sheets held in place by HA.

### Impact of ASC sheet treatment on tendon histology

FDP tendon histology was evaluated on H&E-stained sections from the three repair groups (*N* = 4–5 per group). For semiquantitative analysis, each tendon section was divided into three regions relative to the site of tendon incision (proximal, center, and distal) and evaluated for overall cellularity, vascularity, and relative abundances of PMNs, MNs, and FBs at the tendon surface of each region using a scoring system described in the Methods section (Table 3). Among the three regions evaluated, only the proximal regions (adjacent to the blood supply provided by vincula) contained obvious vasculature, and these regions were also enriched in FBs (Table 4). As anticipated, the application of autologous ASCs to the tendon surface increased the number of FBs at the distal surface. The treatment, however, did not increase the number of FBs at the surface of the repair center and in the regions proximal to the repair (Table 4). ASCs implanted at these regions likely migrated away from the surface to the deep tendon substance, as observed in GFP-expressing tendon sections (Fig. 2e, f).

**Table 4:** Average histological scores of flexor tendons at proximal, center, and distal regions of repair site

	Distal					Center					Proximal				
Group	Cellularity	PMN	MN	FB	Vascularity	Cellularity	PMN	MN	FB	Vascularity	Cellularity	PMN	MN	FB	Vascularity
Repair only	1	0	1	0.5	0	1	1	1	1.5	0	2	1	1	2	2
+HA	1	0	1	0	0	1	1	1	0.5	0	2	1	1	2	2
+ASC sheet	1	1	1	1	0	1	1	1	0.5	0	2	1	1	2	2

*PMN* polymorphonuclear cell, *MN* mononuclear cell and macrophage, *FB* fibroblast cell, *HA* hyaluronan, *ASC* adipose-derived mesenchymal stromal cell

### Effect of ASC sheet treatment on macrophage polarization

**Fig. 3:**
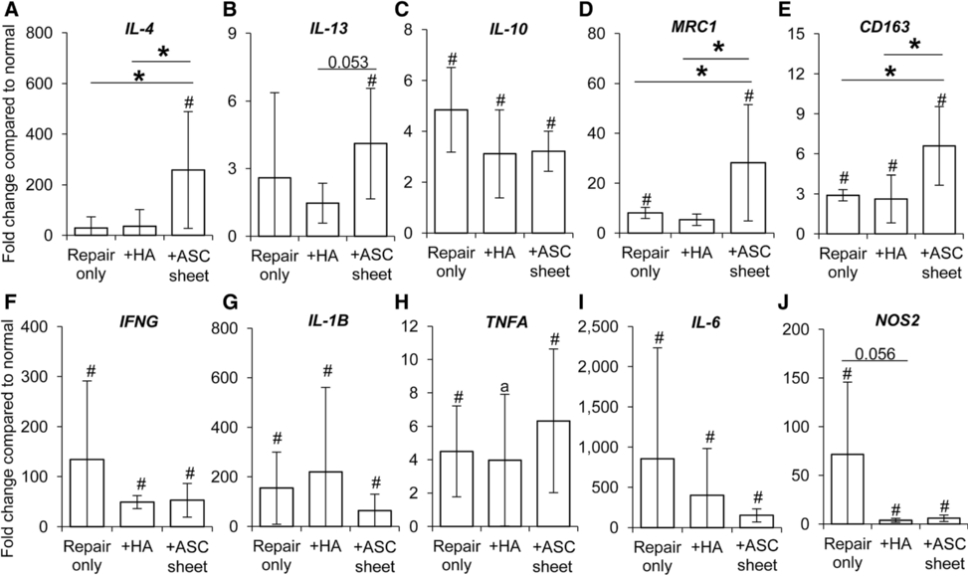
Change in expression of genes associated with macrophage polarization in FDP tendons 7 days post operatively. M2 stimulator genes: **a** *IL-4*, **b** *IL-13*, and **c** *IL-10*. M2 marker genes: **d** *MRC1* and **e** *CD163*. M1 stimulator genes: **f** *IFNG*, **g** *IL-1B*, **h** *TNFA*, and **i** *IL-6*. M1 marker: **j** *NOS2.* *P <0.05 between indicated groups. ^#^ *P* < 0.05 and ^a^ *P* < 0.095, respectively, compared with normal tendons. *HA* hyaluronan, *ASC* adipose-derived mesenchymal stromal cell

**Fig. 4:**
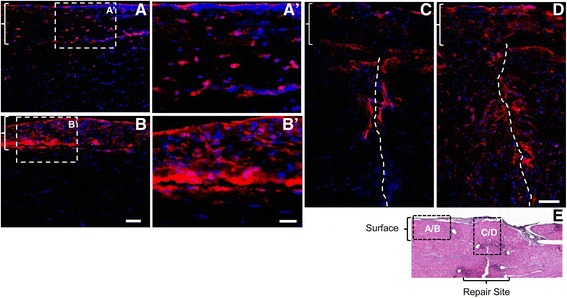
Impact of ASC sheets on M2 macrophage phenotype in FDP tendons 7 days after repair. Representative immunofluorescence images of FDP tendon sections from the + HA (*A*, *A*′, and *C*) or + ASC sheet (*B*, *B*′, and *D*) groups at the tendon surface (*A*, *A*′, *B*, and *B*′) or repair site (*C* and *D*). All sections were stained with M2 macrophage marker CD163 (*red*) and DAPI nuclear stain (*blue*). *E* Location index indicating the location of *A–D* relative to the tendon repair in a tendon section stained with H&E. *A–D Braces* indicate epitenon regions. *C, D Dotted lines* indicate site of tendon transection. *Bar* = 100 μm for *A* and *B*, *bar* = 50 for *A′* and *B′*, and *bar* = 100 μm for *C* and *D*

In light of the potential of ASCs to modulate macrophage phenotype [15], we examined the expression levels of the M2 stimulator genes *IL-4*, *IL-13*, and *IL-10* and the M2 marker genes *MRC1* and *CD163* in FDP tendons from the normal, repair only, + HA, and + ASC sheet groups (*N* = 5 per group). The results showed that ASC treatment significantly increased *IL-4* and *IL-13* expression by 258-fold and 4-fold, respectively (Fig. 3a, b). In contrast, no significant changes in these genes were detected in the tendons from the + HA and repair only groups (Fig. 3a, b). *IL-10* expression was universally increased in tendons from all repair groups, without apparent group differences (Fig. 3c). The expression of M2 marker genes *MRC1* and *CD163* was higher in ASC-treated tendons than in tendons from the other repair groups (Fig. 3d, e). Although the expression levels of M1 stimulator genes *IFNG*, *IL-1B*, *TNFA*, and *IL-6* and marker *NOS2* were increased after FDP tendon repair, there were no significant differences in expression levels among the three repair groups, except for *NOS2* (Fig. 3f–j). There was a trend for reduced expression of this M1 marker gene after either HA or ASC treatment. Consistent with the gene expression results, immunostaining demonstrated CD163^+^ cells at the tendon surface (braced regions in Fig. 4a–d) and at the repair site (dotted lines in Fig. 4c, d). ASC sheet-treated repairs had increased numbers of CD163^+^ cells in both regions compared with + HA repairs (Fig. 4). Together, these results support a role of ASC sheet in promotion of the M2 phenotype during the early stages of tendon healing.

### Effect of ASC sheet treatment on downstream factors related to macrophage activation

**Fig. 5:**
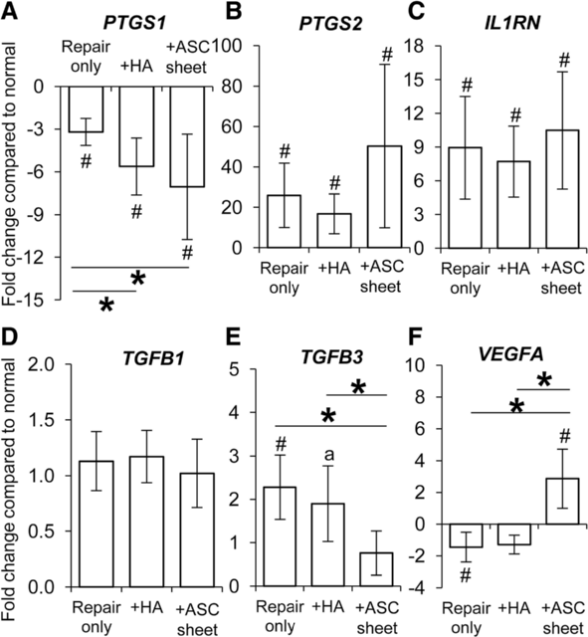
Change in expression of genes associated with downstream effects following macrophage activation in FDP tendons 7 days post operatively. **a** *PTGS1*, **b** *PTGS*2, **c** *IL1RN*, **d** *TGFB1*, **e** *TGFB3*, **f** *VEGFA*. *P < 0.05 between indicated groups. ^a^ *P* = 0.062 and ^#^ *P* < 0.05, respectively, compared with normal tendons. *HA* hyaluronan, *ASC* adipose-derived mesenchymal stromal cell

M2 macrophages have been reported to modulate inflammation, tissue repair, and angiogenesis in a variety of tissues [11]. Therefore, we examined the expression of M2-related genes in FDP tendons from the normal, repair only, + HA, and + ASC sheet groups (*N* = 5 per group). Although *PTGS*2 (encoding cyclooxygenase 2 (COX2)) expression was significantly increased in all repair groups (Fig. 5b), *PTGS1* (encoding COX1) was noticeably reduced after suture repair (Fig. 5a), and ASC sheet treatment further reduced the *PTGS1* level by over 80 % (Fig. 5a). *IL1RN* (encoding IL-1RA) was increased in all repair groups (Fig. 5c). While *TGFB1* expression changed minimally in tendons from any of the repair groups (Fig. 5d), *TGFB3* was moderately increased after suture repair (Fig. 5e). ASC sheets prevented much of the postoperative increase of *TGFB3* in the repaired tendons. ASC sheet treatment increased expression of the angiogenic factor *VEGFA* in FDP tendons, which was significantly reduced after suture repair compared with normal (Fig. 5f). Thus, consistent with M2 macrophage activities, ASC sheets positively modulated tendon responses following intrasynovial tendon repair.

### Effect of ASC sheet treatment on tendon matrix formation and remodeling

**Fig. 6:**
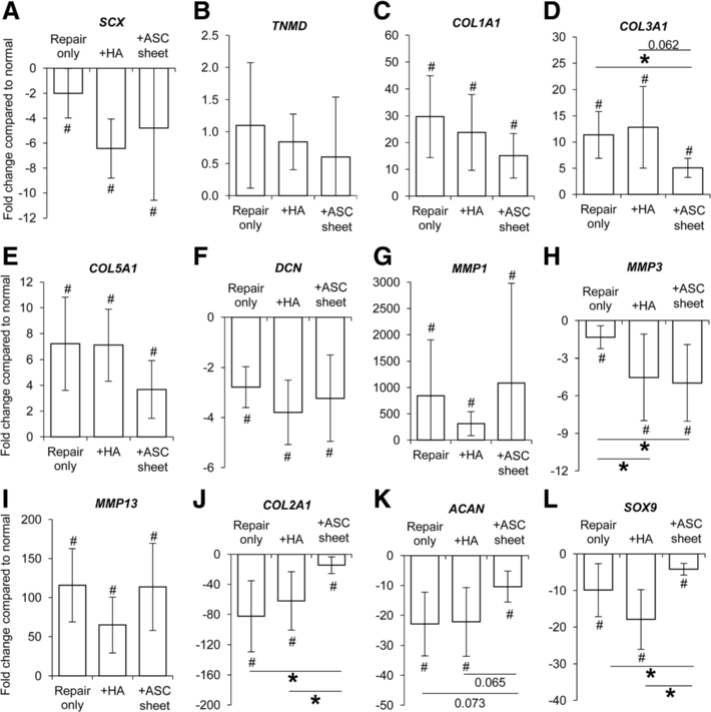
Change in expression of tendon-related genes in FDP tendons 7 days after repair. **a** *SCX*, **b** *TNMD*, **c** *COL1A1*, **d** *COL3A1*, **e** *COL5A1*, **f** *DCN*, **g** *MMP1*, **h** *MMP3*, **i** *MMP13*, **j** *COL2A1*, **k** *ACAN*, **l** *SOX9*. *P < 0.05 between indicated groups. ^#^ *P* < 0.05 compared with normal tendons. *HA* hyaluronan, *ASC* adipose-derived mesenchymal stromal cell

Because MSCs and M2 macrophages are known to promote tissue growth and matrix remodeling [21, 22], we examined the expression of genes related to TF phenotype and tendon matrix formation and remodeling. Compared with normal tendons, tendon injury and repair led to significant changes in all genes investigated (except *TNMD*) in each of the three repair groups (Fig. 6). Although the tenogenic transcription factor *SCX* (Fig. 6a) and the tendon matrix gene *DCN* (Fig. 6f) were both significantly reduced in the repaired tendons compared with normal, the expression of tendon matrix genes *COL1A1*, *COL3A1*, and *COL5A1* were dramatically increased (Fig. 6c–e). When examining MMP gene expression, *MMP1* (Fig. 6g) and *MMP13* (Fig. 6i) were increased and *MMP3* (Fig. 6h) was decreased in all repair groups compared with normal.

ASC sheet treatment led to a reduction in the scar-associated gene *COL3A1* (Fig. 6d). Expression of *MMP3* was further reduced in the + ASC sheet group compared with the repair only group (Fig. 6h); however, this effect may not have been specific to ASC sheet treatment, because a similar change was detected in the + HA group without an ASC sheet. As flexor tendons are subjected to compression loads at a number of locations along their length, their extracellular matrix is often enriched in cartilage-associated matrix [23]. Therefore, we evaluated the expression of cartilage matrix genes *COL2A1* and *ACAN* and the chondrogenic gene *SOX9*. Tendon injury dramatically reduced the expression of these three genes in all repair groups. In contrast, ASC sheet treatment led to recovery of *COL2A1* expression to levels similar to normal tendons (Fig. 6j). Similarly, the reduction of *ACAN* expression in repaired tendons was mitigated by ASC treatment (Fig. 6k). Likewise, *SOX9* expression in the ASC sheet-repaired tendons was significantly greater than that noted in the other two repair groups (Fig. 6l). Taken together, results demonstrated the potential of an ASC sheet to regulate tendon matrix formation and remodeling during tendon healing.

### Effect of ASC sheet treatment on tendon cell apoptosis

**Fig. 7:**
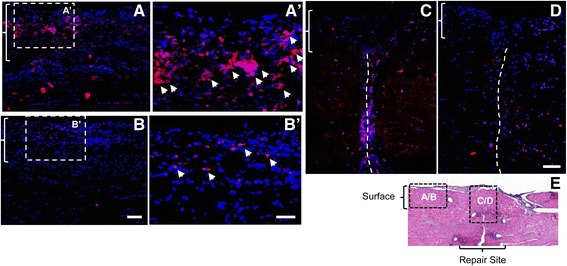
Impact of ASC sheets on tendon cell apoptosis in FDP tendons 7 days after repair. Representative immunofluorescence images of FDP tendon sections from the + HA (*A*, *A*′, and *C*) or + ASC sheet (*B*, *B*′, and *D*) groups at the tendon surface (*A*, *A*′, *B*, and *B*′) or repair site (*C* and *D*). All sections were stained for cleaved caspase 3 (*red*) and DAPI nuclear stain (*blue*). *E* Location index indicating the location of *A*–*D* relative to tendon repair in a tendon section with H&E staining. *A–D Braces* indicate epitenon regions. *C, D Dotted lines* indicate site of tendon transection. Bar = 100 μm for *A* and *B*, *bar* = 50 μm for *A′* and *B*′, and bar = 100 μm for *C* and *D*

M1 macrophage activation has been linked to apoptotic cell death [10, 15], and cell apoptosis at the flexor tendon repair site may contribute to adhesion formation and affect the accrual of strength during healing [24, 25]. Therefore, we tested the ability of ASC sheet treatment, presumably through promotion of the M2 macrophage phenotype, to protect tendon cells from apoptotic cell death following repair. Cleaved caspase 3 immunostaining, which marks apoptotic cells [26], was performed on tendon sections from the + HA and + ASC sheet groups (*N* = 3 per group). Cleaved caspase 3 positive signal (in red) primarily accumulated at the tendon surface (Fig. 7a, b) and at the repair site (Fig. 7c, d). ASC treatment (Fig. 7b, b′, d) reduced cleaved caspase 3 signals in both regions.

## Discussion

Repaired intrasynovial flexor tendons are susceptible to gap formation and rupture during the first 3 weeks following suture, and to the formation of adhesions between the tendon and its surrounding synovial sheath [1–5]. Although low levels of inflammatory cytokines are likely necessary to attract FBs to the repair site [5, 6], a marked inflammatory response after tendon suture has been identified as a key factor leading to highly variable clinical outcomes [6, 8, 15]. Control of the inflammatory environment after operative repair is therefore a potential therapeutic target. In the current study, we demonstrated that the delivery of ASCs to the surface of a repaired tendon via a new ASC sheet approach was effective in modulating the inflammatory environment in the early period after repair. Because recent evidence suggests that fine modulation of inflammation in the earliest stages following operative repair can improve healing [12, 15, 16, 27], the approach presented here holds great promise for enhancing flexor tendon repair.

The findings in the current study are consistent with a prior in-vitro report demonstrating that ASCs suppress the negative effects of macrophages on TFs by inducing a phenotypic switch from the proinflammatory M1 macrophage phenotype to the anti-inflammatory M2 macrophage phenotype [15]. Prior in vitro and in vivo studies in other tissues have also shown that MSCs have the capacity to alter the inflammatory environment and improve the healing response [16, 27–29]. However, it remains unclear how ASCs regulate inflammation during tendon healing. ASCs may secrete factors that modulate macrophage activity by promoting changes in macrophage differentiation or cytokine expression patterns, may secrete factors that modulate TF activity leading to reduced sensitivity to cytokines such as IL-1B, and/or may inactivate circulating proinflammatory factors (e.g., by releasing factors that degrade or sequester circulating proinflammatory cytokines). The results of the current study support the premise that the primary effect of ASC application is modulation of macrophage differentiation. The application of ASCs in vivo led to increased expression of the M2 stimulator genes *IL-4* and *IL-13* and the M2 marker genes *CD163* and *MRC1*. This concept is supported further by the histological results, which demonstrated that implanted ASCs accumulated at the tendon surface and within the repair site, where infiltrating monocytes first arrived and CD163^+^ cells were initially detected. The coappearance of ASCs and macrophages indicates a likely interaction between the two cell types. Previous reports have indicated that the interaction may be mediated via a paracrine mechanism [30]. Nevertheless, the underlining mechanisms are still unclear. Besides macrophages, ASCs may have﻿ also ﻿influenced the function of other neighboring cells by modulating the local molecular environment.

The functional significance of the M2 phenotype is its potential to improve tendon healing by promoting angiogenesis, cell proliferation, and matrix remodeling while concurrently suppressing cell death and tissue damage [11, 15, 28]. Consistently, the current study revealed that ASC treatment promoted *VEGF* expression, retained expression of tendon matrix genes *COL2A1* and *COL3A1* levels closer to the levels found in the healthy tissue, and suppressed tendon cell apoptosis. The ability of ASCs to promote the M2 phenotype and modulate the inflammatory environment may be influenced by factors such as the magnitude and chronicity of the injury, the age of patient, and the source of the cells. However, it has been shown that the potency of ASCs can also be modified by exogenous stimuli [31], thus broadening the potential applications of ASCs to multiple tendon pathologies and patient populations. Longer time points of healing are necessary, however, to determine whether these findings will result in functional improvements.

Despite the apparent induction of M2 macrophages by ASCs, there was no significant reduction in major proinflammatory cytokine genes (e.g., *IL-1B* and *IFNG*) in the current study. This discrepancy may have resulted from a variation in individual responses to injury and ASCs, as indicated by the relatively high standard deviations seen in the gene expression results. In addition, given the massive scale of initial inflammatory responses after tendon repair [6], modulation of the natural response may require a longer interval. In prior studies, a suppressive effect of ASCs on *IL-1B* expression was undetectable until 5 days after ASC–macrophage coculture [15]. Because both implanted ASCs and macrophages need to migrate to the repair site, it may take even longer time to detect this effect in vivo*.*

A major challenge in biological enhancement of intrasynovial tendon healing is the necessity to stimulate extracellular matrix formation at the repair site (to improve tendon strength) while at the same time suppressing extracellular matrix formation at the tendon surface (to allow for gliding). Delivery of cells and/or growth factors to repaired tendons can stimulate adhesions if they are not localized to the repair site and effectively isolated from the intrasynovial space [9]. The current study explores a new solution to this problem. ASCs were formed into sheets and wrapped around the tendon at the repair site. To prevent stimulation of adhesions and to anchor the ASC sheet to the tendon, a thin layer of HA hydrogel was applied superficially to the ASC sheet. The approach resulted in viable ASCs infiltrating the tendon repair site by 7 days post operation. Of importance, there were no adhesions noted in any of the treated repairs. This is in contrast to a recent study where ASCs and the growth factor BMP-12 were implanted in the interior of the tendon via surgically generated slits [8]. In that study, a low-grade inflammatory reaction developed due to the polymer-based scaffold, negating any positive effects from the treatment. Future studies using the new cell sheet platform will incorporate growth factors along with stem cells and will test the potential to improve both functional and structural outcomes.

There were a number of limitations to the current study. First, due to the study’s focus on demonstrating the feasibility of a new approach and the emphasis on the early inflammatory processes, only one early time point was examined. Although the ASCs were shown to influence the inflammatory response, it is not known whether this modulation will result in longer-term functional benefits. However, now that the novel method has been established as safe and effective in the short term, the efficacy of the approach can be explored further in longer-term studies. Second, gene expression results do not always correlate with protein expression levels and activities. However, current immunohistochemical results strongly supported one of the primary gene expression outcomes of the study: the M2 phenotypic marker CD163 was significantly increased in the ASC-treated group compared with control. This result is strengthened by prior proteomics data from healing FDP tendons, which confirmed the substantial accumulation of CD163 proteins after ASC treatment [8]. Third, the mechanisms by which ASCs promote the M2 phenotype were not explored in the current study. Furthermore, M1 and M2 phenotypes lie at the extremes of a continuum of macrophage expression profiles, and it remains unclear whether certain M1 characteristics remain after ASC treatment.

## Conclusions

In summary, a cell sheet approach was used to deliver autologous ASCs to the surface of repaired flexor tendons in a clinically relevant large animal model. The ASC sheets modulated the inflammatory phase of tendon healing, as demonstrated by gene expression and immunohistological assessment 7 days post repair. Specifically, ASCs promoted a regenerative/anti-inflammatory M2 macrophage phenotype and influenced tendon extracellular matrix remodeling, angiogenesis, and cell survival. Future studies will explore the potential for this approach to enhance functional outcomes after flexor tendon repair.

## Abbreviations

ANOVA: Analysis of variance
ASC: Adipose-derived mesenchymal stromal cell
COX: Cyclooxygenase
FB: Fibroblast
FDP: Flexor digitorum profundus
GFP: Green fluorescent protein
HA: Hyaluronan
H&E: Hematoxylin and eosin
HPF: High-power field
IL-1RA: IL-1 receptor antagonist
M1: Type 1 macrophage phenotype
M2: Type 2 macrophage phenotype
MN: Mononuclear cell
MSC: Mesenchymal stromal cells
PBS: Phosphate-buffered saline
PFA: Paraformaldehyde
PI: Propidium iodide
PMN: Polymorphonuclear cell
TF: Tendon fibroblast
